# Long-term Cumulative Incidence of Clinically Diagnosed Retinopathy in the Finnish Diabetes Prevention Study

**DOI:** 10.1210/clinem/dgae287

**Published:** 2024-04-25

**Authors:** Kai Kaarniranta, Mikko Valtanen, Sirkka Keinänen-Kiukaanniemi, Jaakko Tuomilehto, Jaana Lindström, Matti Uusitupa

**Affiliations:** Institute of Medical Sciences, School of Medicine, University of Eastern Finland, 70211 Kuopio, Finland; Department of Ophthalmology, Kuopio University Hospital, 70029, Kuopio, Finland; Department of Molecular Genetics, Faculty of Biology and Environmental Protection, University of Lodz, 90-236 Lodz, Poland; Department of Chronic Disease Prevention, National Institute for Health and Welfare, 00271 Helsinki, Finland; Center for Life Course Health Research, University of Oulu, 90014 Oulu, Finland; Department of Chronic Disease Prevention, National Institute for Health and Welfare, 00271 Helsinki, Finland; Department of Public Health, University of Helsinki, 00014 Helsinki, Finland; Saudi Diabetes Research Group, King Abdulaziz University, 21589 Jeddah, Saudi Arabia; Department of Chronic Disease Prevention, National Institute for Health and Welfare, 00271 Helsinki, Finland; School of Medicine, Institute of Public Health and Clinical Nutrition, University of Eastern Finland, 70211 Kuopio, Finland

**Keywords:** diabetes, glucose, HbA_1c_, lifestyle intervention, prevention, retinopathy

## Abstract

**Context:**

Lifestyle intervention reduces the incidence of type 2 diabetes (T2D) in people with impaired glucose tolerance (IGT).

**Objective:**

This work aimed to find out whether participation in an earlier lifestyle intervention had an effect on the occurrence of clinically diagnosed diabetic retinopathy (DR) during a median of 22 years of follow-up time.

**Methods:**

The study included 505 individuals from the Finnish Diabetes Prevention Study (DPS) (mean age 55; range, 40-64 years at the onset of the study) with IGT who were originally randomly assigned to the intervention (weight loss, healthy diet, and physical activity) (N = 257) and usual care control groups (N = 248). The median follow-up was 22 years. Clinical retinopathy diagnoses were obtained from the Finnish national hospital Care Register for Health. Data on glycemic parameters, serum lipids, and blood pressure were available from both the intervention (median 4 years) and postintervention period (until year 7).

**Results:**

No significant difference was found in the cumulative incidence of clinically diagnosed DR between the original intervention (N = 23, 8.9%) and control groups (N = 19, 7.7%) during the extended follow-up (odds ratio: 1.15; 95% CI, 0.61-2.21). A higher cumulative glycated hemoglobin A_1c_ (HbA_1c_) was significantly associated with a higher risk of retinopathy (hazard ratio 1.4; 1.02-1.88, 95% posterior interval, adjusted for group, age, and sex). Furthermore, the incidence of retinopathy diagnosis was numerically more common among individuals who had developed diabetes during the follow-up (33/349) compared with those who had not (9/156); however, the comparison was not statistically significant (odds ratio: 1.86, 95% CI, 0.89-4.28, adjusted for group, age, and sex).

**Conclusion:**

A higher cumulative HbA_1c_ was significantly associated with a higher risk of retinopathy. No evidence was found for a beneficial effect of a 4-year lifestyle intervention on the long-term occurrence of clinical DR during a median of 22-year follow-up.

Diabetic retinopathy (DR) is the most common diabetic microvascular complication (1). Currently, approximately 103 million individuals in the world suffer from DR which is the leading cause of vision loss in working-age individuals (1). DR is strongly associated with poor glycemic control, long duration of diabetes, hypertension, and dyslipidemia (2, 3). It is also associated with other late chronic complications of diabetes, such as diabetic kidney disease and cardiovascular disease (4). Microvascular complications of the retina constitute microaneurysms (MAs), hemorrhages, hard exudates and interretinal microvascular abnormalities, macular edema, and proliferative vessel growth (5). Such lesions result in progressive retinal disease and even permanent vision loss if not treated properly (6). In people with diabetes, good glycemic control has a major effect on reducing the development and slowing the progression of DR (3, 6).

Several studies have demonstrated that lifestyle interventions aimed at permanent weight reduction, increased physical activity, and healthy diet can result in a more than 50% reduction in diabetes incidence in adults with impaired glucose tolerance (IGT) followed for 1 to 6 years (7, 8). Interestingly, DR signs such as MAs can be observed in individuals with prediabetes (9–12). Recent results from the Diabetes Prevention Program Outcome Study (20 years after random assignment into the Diabetes Prevention Program) showed no difference in DR between the original lifestyle intervention and control groups but the prevalence of DR was higher in the people who had developed T2D (24%) than in those who remained without diabetes (14%) (13). In the Finnish Diabetes Prevention Study (DPS) (14), in individuals with IGT the lower risk of developing T2D was associated with a higher adherence to lifestyle changes and more marked improvement of insulin sensitivity and dyslipidemia (14–17). Furthermore, a lower prevalence of retinal microvascular abnormalities (MAs) was found in the intervention group (11). Besides glycemia, higher serum triglyceride level was associated with early DR abnormalities. Furthermore, n-3 long-chain unsaturated fatty acids, odd-chain fatty acid 15:0, and plasmalogen dm16:0 were associated with a lower risk of MAs (18). In the present study, we analyzed the prevalence of clinically diagnosed DR during the 22-year median follow-up in the former DPS study participants.

## Materials and Methods

**Figure 1.**
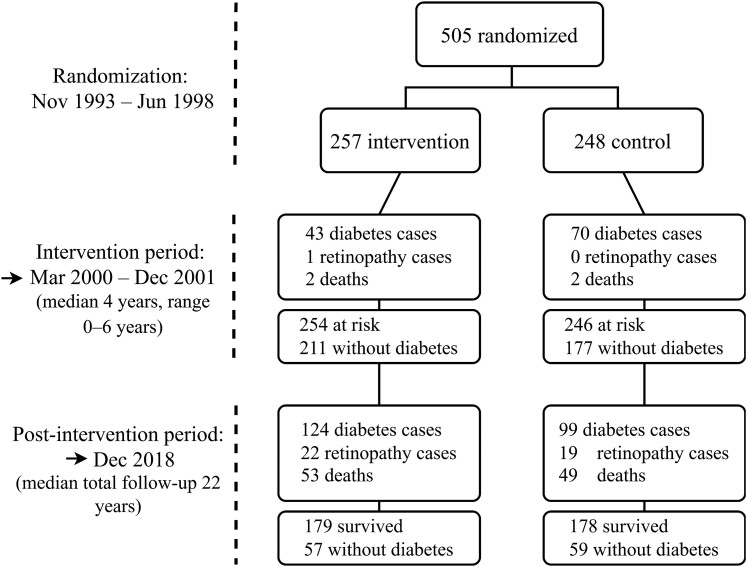
Flowchart of the Diabetes Prevention Study follow-up study.

A total of 522 people with IGT were recruited into the multicenter-controlled DPS study in Finland during 1993 to 1998 (Fig. 1 and Table 1). The median intervention period lasted for 4 years and was then followed by a nonintervention follow-up lasting up to 11 years (16). The current results on the occurrence of clinically diagnosed retinopathy are based on the median total follow-up of 22 years. Originally, 265 people were randomly assigned to the intervention group, which received an intensive lifestyle intervention in terms of weight loss, healthy dietary choices, and increasing physical activity, whereas 257 people served as controls who received general instruction for a healthy lifestyle (14–16). In this follow-up study, we had data on 257 and 248 individuals from the original intervention and control groups, respectively, who gave consent for record linkage with national health registries.

**Table 1.** Baseline characteristics and follow-up data on antihypertensive and lipid and glucose-lowering drug treatment and self-reported physical activity at baseline and follow-up

	Intervention group	Control group
No. (men/women)	257 (88/169)	248 (78/170)
Follow-up time,	22.3	22.2
median (interquartile range), y	(21.6-23.4)	(21.8-22.9)
Age, y	55 (7.3)	55 (6.9)
Body mass index	Men 30.1 (3.6)	Men 29.8 (3.6)
	Women 32.1 (4.9)	Women 31.8 (4.7)
Fasting plasma glucose, mmol/L	6.1 (0.8)	6.2 (0.7)
2-h plasma glucose, mmol/L	8.9 (1.5)	8.9 (1.5)
HbA_1c_, %	5.7 (0.6)	5.6 (0.6)
Serum total cholesterol, mmol/L	5.6 (1.0)	5.6 (0.9)
HDL cholesterol, mmol/L	Men 1.1 (0.3)	Men 1.1 (0.3)
	Women 1.3 (0.3)	Women 1.3 (0.3)
Serum triglycerides, mmol/L	1.7 (0.8)	1.8 (0.8)
Systolic blood pressure, mm Hg	140 (18)	136 (17)
Diastolic blood pressure, mm Hg	86 (9.4)	86 (10)
Drug treatment for hypertension (%), baseline	71 (28%)	78 (31%)
Last visit during follow-up	155 (60%)	146 (59%)
Lipid-lowering drug treatment (%), baseline	11 (4%)	15 (6%)
Last visit during follow-up	113 (44%)	109 (44%)
Regular smokers, baseline (%)	14 (5%)	18 (7%)
Reported physical*^a^* activity, baseline (%)	162 (63%)	164 (66%)
Last visit during follow-up	167 (65%)	160 (65%)
Diabetes diagnosis during follow-up	167 (65%)	169 (68%)

Abbreviations: HbA_1c_, glycated hemoglobin A_1c_; HDL, high-density lipoprotein.

^*a*^At least 4 hours per week walking, bicycling, running, or other moderate intensity physical activity per week.

We have previously reported early retinopathic changes based on fundus photographs in a subsample of the DPS study participants (11, 18). Detailed description of the methods, clinical characteristics, physiological, and biochemical measurements have been given in previous publications (14–16, 19). In brief, all participants had an annual oral glucose tolerance test, a medical history, and physical examination with measurements of height (without shoes), weight (in light indoor clothes), waist circumference (midway between the lowest rib and iliac crest), and blood pressure. Serum total cholesterol, high-density lipoprotein (HDL) cholesterol, and triglycerides were determined from fasting samples using an enzymatic assay method. In the postintervention period until the last clinic visit, biannual examinations were carried out (16).

For the present postintervention study, the data on clinically diagnosed DR were collected through computerized register linkage to 2 nationwide health registers: the Care Register for Health and the Causes of Death Register, using the national personal identification number (ID). The DR cases were identified by searching the databases up until a predefined end-of-study date, December 31, 2018, for International Classification of Diseases, Tenth Revision (ICD-10) codes with the following prefixes: E10.3, E11.3, E12.3, E13.3, E14.3, H28.0, and H36.0; or CKC12/CKD05/CKD60/CKD65. New diabetes cases were diagnosed during the clinical study visits by a 2-hour oral glucose tolerance test, which was used as the primary source for DM diagnoses. After an individual's last clinical visit, the search for DM diagnoses was continued through the Care Register for Health and the registers for Drug Reimbursements and Drug Purchases for ICD-10 codes starting with E11 or Anatomical Therapeutic Chemical (ATC) codes starting with A10A or A10B (excluding A10BX01). A diagnosis was then declared on the date of the first register entry indicating diabetes if an individual had either i) at least one (Drug Reimbursement code) or ii) 2 or more total register entries indicating DM. Data from these registers were also available for deceased individuals before death. In Finland, all residents have a personal ID. This number is used in all health registers covering all hospital admissions in the country. Using this ID number, we could identify all hospital admissions of the DPS participants. Deaths and clinically diagnosed retinopathy cases are based on a median of 22 years’ follow-up time, which means there are no missing registry data for ID-coded individuals.

The study protocol was approved by the ethics committees of the National Public Health Institute in Helsinki, Finland (intervention phase), and of the North Ostrobothnia Hospital District (follow-up period). All study participants gave written informed consent at baseline and again at the beginning of the postintervention follow-up.

### Statistical Analyses

Logistic regression models were used to assess whether the assignment to lifestyle intervention or getting a diabetes diagnosis during follow-up was associated with getting a retinopathy diagnosis during the entire follow-up, that is, the time from baseline to death or end-of-study date, defined here as December 31, 2018. Models assessing the possible contribution of the diabetes diagnosis were adjusted for the intervention group status, sex, age at baseline, and follow-up time. Two models were fitted separately: one using the diabetes diagnosis at any time during the follow-up as the predictor, and another one using the diagnosis of diabetes during the first 5 years of follow-up as the predictor. The model assessing the effect of the intervention assignment was adjusted for sex, age at baseline, and follow-up time. Individuals who died during the follow-up were also included in the analyses.

Associations between the cumulative risk factor histories (glycated hemoglobin A_1c_ [HbA_1c_], fasting and 1-hour and 2-hour plasma glucose, serum total cholesterol, HDL cholesterol, triglycerides, systolic and diastolic blood pressure, and body mass index) and DR were assessed by joint modeling (20) of the respective longitudinal risk factor trajectory and the hazard for DR. The survival submodels were specified as proportional hazards models. For the longitudinal, linear mixed-effects models were used, and the possibly nonlinear shapes of the risk factor trajectories were accommodated by using natural cubic splines with 1 inner knot placed at a 5-year mark. Prior to the analyses, risk factors were centered to the means of their baseline distributions and scaled to units of their baseline SDs. To capture the cumulative effects the risk factors were assumed to have on the hazard of retinopathy, cumulative parameterization (21, 22) was used to link the 2 submodels, that is, incorporating the integral of risk factor trajectories into the linear predictor of the survival submodel. Both submodels were adjusted for intervention assignment, sex, and age at baseline.

In addition, we conducted sensitivity analysis, by which the individuals were censored after 7 years since their last clinical follow-up visit if not already censored for other reasons. The 7-year cutoff was chosen to limit the required extrapolation of estimated longitudinal trajectories while still retaining most of the clinical end points. The joint models were fitted using R software for statistical computing (version 4.3.0) and package JMbayes2 (version 0.4.5), using package-default priors. The results are presented as hazard ratios, interpretable as the effect associated with a 1-baseline-SD increase of the risk factor for 1 year. In addition, 95% posterior intervals for the estimates are reported, and considered statistically significant if the interval does not contain 1.

## Results

Table 1 summarizes the baseline data of the study participants by group. Use of antihypertensive and lipid-lowering drugs increased markedly in both groups during the follow-up (see Table 1), but no differences between the groups were found in this regard.

**Figure 2.**
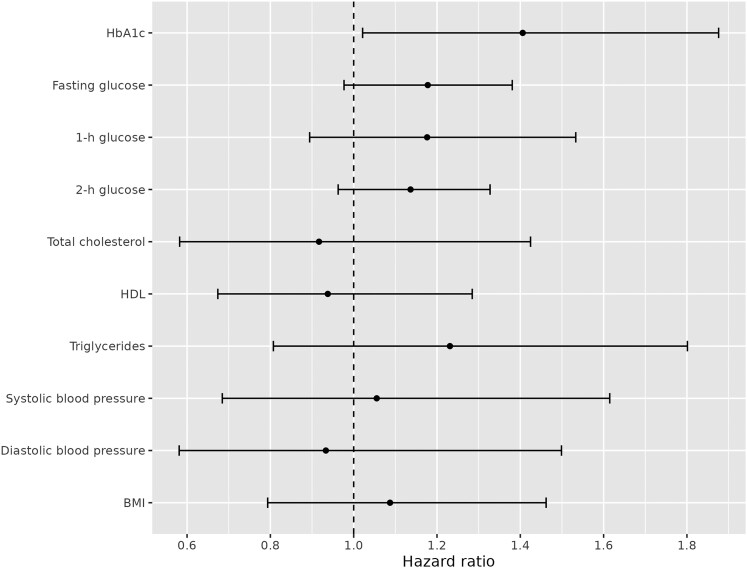
Estimated standardized hazard ratios and 95% posterior intervals associated with a 1-year increase in risk factor by 1 unit during the follow-up. The unit was calculated as 1 SD of the baseline value of the risk factor, and therefore the estimated hazard ratios among risk factors are directly comparable.

Fig. 1 shows the flowchart of the formation of the present study population, cumulative incidence of diabetes and DR, and the number of deaths at the end of intervention period and after the extended follow-up of a median of 22 years. The outcome measures of the present study, that is, diabetes and DR, were based on the data documented in the Finnish health registers with computerized record linkage using the national personal ID resulting in a complete case-ascertainment during the follow-up. No significant difference was observed between the original intervention (N = 23, 8.9%) and control groups (N = 19, 7.7%) in the occurrence of DR (OR 1.15; 95% CI, 0.61-2.21). Next, we analyzed the effects of cumulative longitudinal trajectories of HbA_1c_, fasting and 1-hour and 2-hour plasma glucose, serum lipids, blood pressure, and body mass index on the hazard of DR in the entire study population (Fig. 2). HbA_1c_ was the only risk factor significantly associated with the hazard for DR (hazard ratio 1.4; 95% posterior interval, 1.02-1.88, adjusted for group, age, and sex). The estimates for other glycemic measures showed a similar but smaller association; however, they were not considered significant since the posterior intervals for hazard ratios overlap 1 (Fig. 2). The estimates remained similar in the sensitivity analysis.

Of the individuals who received a diabetes diagnosis at any time during the follow-up, almost 10% (33/349) also received a DR diagnosis, whereas this proportion among those who remained free of diabetes was 6% (9/156) . The estimated odds ratio, adjusted for group, sex, age at baseline, and follow-up time, was 1.81 (95% CI, 0.86-4.19); however, the difference was not statistically significant.

## Discussion

The main finding of the present study was that hyperglycemia revealed by a higher cumulative HbA_1c_ was significantly associated with an increased risk of DR. No evidence was found for an effect of lifestyle intervention on the occurrence of DR during the median of 22 years of follow-up. Furthermore, individuals who had developed diabetes during the follow-up were found to have a numerically higher risk of DR than those remaining free of diabetes (odds ratio 1.81; 95% CI, 0.86-4.19), although the result was not statistically significant. Nevertheless, this can also be taken as evidence for the effect of hyperglycemia.

Our previous report on the DPS follow-up suggested that an intensive lifestyle intervention for 4 years in overweight and obese individuals with IGT could prevent the appearance of retinal MAs (11). Nevertheless, in this long-term follow-up study with a median follow-up of 22 years, we did not find a significant difference in the occurrence of clinically diagnosed DR leading to hospital admission between the original intervention and control groups. Furthermore, we did not observe a significant association between DR and serum triglycerides that we observed in our earlier assessment on the occurrence of retinal MAs based on the fundus photography examination (11). Other lipid metabolites, including serum plasmalogens, were not associated with clinically diagnosed DR, either (18).

Several studies and meta-analyses have confirmed that T2D can be prevented or postponed in prediabetic individuals by lifestyle modification, pharmacological interventions, or a combined intervention (8, 23, 24). Only a few studies have examined whether lifestyle intervention in prediabetes could also prevent the development of DR (Table 2). Lifestyle intervention lasting 6 years in people with IGT in the Chinese Da Qing Diabetes Prevention Outcome Study delayed the onset of T2D and also reduced the incidence of cardiovascular events, microvascular complications, and cardiovascular and all-cause mortality, and increased life expectancy (25–27). Long-term results of this Chinese study showed a cumulative incidence of DR 13.5% in combined intervention groups and 30.7% in a control group over a 30-year interval (25). The reduced incidence of diabetes in the intervention group was suggested to be the main reason for the decreased DR in this Chinese study. In the Spanish PREDIMET study, the 6-year incidence of DR was only 2%, but it was 44% lower in the extra virgin olive oil group than in the control group (28). On the other hand, the US Diabetes Prevention Program Outcome Study showed that lifestyle intervention as such did not decrease the risk of retinal complications, but the prevalence of DR was 14% in the nondiabetic group and 24% in the group with incident diabetes in that study, that is, those who developed diabetes had an increased prevalence of DR (13, 29; see Table 2). Our results on the prevalence of DR (8.9% in the intervention and 7.7% in the control group), are well in line with those of the Diabetes Prevention Program Outcome Study. The lower prevalence in the DPS study participants may be due to different methods applied to define the occurrence of DR. It is well known that DR may occur in adults with prediabetes and early T2D (11-13; see Table 2). Long-term hyperglycemia measured by elevated HbA_1c_ was a key risk factor for the development of DR across the entire glycemic range from prediabetes to diabetes in the Diabetes Prevention Program Outcome Study. Furthermore, weight and history of hypertension, dyslipidemia, and smoking were associated with DR in that study (13). Danish data revealed that HbA_1c_ was the most important factor in the progression of nonproliferative DR to proliferative DR (30). On the contrary, systolic and diastolic blood pressure or serum cholesterol did not show marked effects on DR progression (30), which is in line with our follow-up data. Furthermore, intensive treatment of hypertension and lipid disorders in the DPS participants may have modified the effect of elevated blood pressure and dyslipidemias on the risk of DR.

**Table 2.** Summary of previously published long-term follow-up of lifestyle intervention studies on diabetic retinopathy in high-risk people with diabetes or individuals with prediabetes

Study	Intervention	Effect on retinopathy	Comments
China Da Qing Diabetes Prevention Follow-up study (26)	Diet clinics, exercise clinics, diet and exercise clinics, and control clinics	40% reduction in any retinopathy in combined intervention clinics compared to original control clinics group	Randomization by clinics, mean follow-up time of 30 y after randomization
Diabetes Prevention Program Outcome Study (29)	Intensive lifestyle support, metformin, and control groups	No group differences in prevalence of any retinopathy	Fundus photographs examination data. Fewer microvascular complications in individuals who remained nondiabetic during follow-up (RR 0.72; *P* < 0.001).
Diabetes Prevention Program DPP Outcome study (13)	See previously mentioned original study groups	Prevalence of retinopathy smaller in individuals without diabetes (14%) than in those with incident diabetes (24%) after 16 y of follow-up period. No difference in any retinopathy between original study groups (lifestyle, metformin, placebo)	Main risk factor for retinopathy was glycemia/diabetes. OR for HbA_1c_ was 1.65 (1.48-1.83, *P* < .0001 per SD, 0.7%) after adjustments. Interestingly, less retinopathy in American Indian individuals
The Finnish Diabetes Prevention Follow-up Study (11)	Lifestyle (diet and exercise) intervention vs control group	Less early retinopathy in intervention than in control groups (24% vs 38%, adjusted OR 0.52; 0.28-0.97, 95% CI; *P* = .039).	Subgroup analysis based on retinal photographs
PREDIMET Study, Mediterranean Diet, Retinopathy, Nephropathy, and Microvascular Diabetes Complications: A Post Hoc Analysis of a Randomized Trial (28)	Mediterranean diet with EVOO (N = 1282) or with nuts (N = 1142) vs control group (N = 1190	Multivariable-adjusted HRs for diabetic retinopathy were 0.56 (95% CI, 0.32-0.97) for MedDiet + EVOO and 0.63 (0.35-1.11) for MedDiet + Nuts compared to control diet group	Post hoc analysis in patients with diabetes. Incidence of retinopathy based on ophthalmological examination or photocoagulation therapy After 6 y of follow-up, incidence of any retinopathy was only 2% in whole study group

Abbreviations: EVOO, extra virgin olive oil; HbA_1c_, glycated hemoglobin A_1c_; HR, hazard ratio; OR, odds ratio; RR, relative risk.

We can only speculate that the preventive effects of n-3 long-chain unsaturated fatty acids and plasmalogens may have a role in earlier phases of MA formation (11, 18) since we did not find any association in the present study between these biomarkers and clinical DR diagnosed mostly in specialized hospital clinics.

Strengths of the study are the well-controlled DPS population with repeated follow-up assessments of conventional risk factors, glycemia, and a multitude of blood biomarkers. The national Care Register for Health data checked every year provided accurate diagnoses from health centers, hospitals, and other institutions with a complete case ascertainment. Our study does have weaknesses. The sample size was markedly lower than in the Diabetes Prevention Program Outcome Study (13) but was comparable to that in the Chinese Da Qing Follow-up study (25–27). We did not have baseline data on retinal changes, either. Formal power calculations concerning DR were not performed; however, as the number of DR events was relatively small, and the CIs relatively wide, null results should not be taken as evidence of absence of a clinically meaningful association.

In conclusion, based mostly on the Care Register for Health data, we confirm that in individuals with IGT a longitudinally good glycemic control and remaining free of diabetes for a long period of time are the key elements to prevent DR in prediabetic individuals. We did not observe that lifestyle intervention in the DPS study as such affected the risk of clinically diagnosed DR that was based on the Care Register for Health data.

## Data Availability

Some or all data sets generated during and/or analyzed during the current study are not publicly available but are available from the corresponding author on reasonable request.

## Abbreviations

DPS: Finnish Diabetes Prevention Study
DR: diabetic retinopathy
HbA_1c_: glycated hemoglobin A_1c_
IGT: impaired glucose tolerance
MA: microaneurysm
T2D: type 2 diabetes

## References

[1] Teo ZL, Tham YC, Yu M, et al Global prevalence of diabetic retinopathy and projection of burden through 2045: systematic review and meta-analysis. Ophthalmology. 2021;128(11):1580‐1591. 33940045 10.1016/j.ophtha.2021.04.027

[2] Mohamed Q, Gillies MC, Wong TY. Management of diabetic retinopathy: a systematic review. JAMA. 2007;298:902‐916. 17712074 10.1001/jama.298.8.902

[3] Yau JW, Rogers SL, Kawasaki R, et al Global prevalence and major risk factors of diabetic retinopathy. Diabetes Care. 2012;35(3):556‐564. 22301125 10.2337/dc11-1909 PMC3322721

[4] Penno G, Solini A, Zoppini G, et al Rate and determinants of association between advanced retinopathy and chronic kidney disease in patients with type 2 diabetes: The Renal Insufficiency And Cardiovascular Events (RIACE) Italian multicenter study. Diabetes Care. 2012;35:2317‐2323. 23093684 10.2337/dc12-0628 PMC3476898

[5] Usman TM, Saheed YK, Nsang A, Ajibesin A, Rakshit S. A systematic literature review of machine learning based risk prediction models for diabetic retinopathy progression. Artif Intell Med. 2023;143:102617. 37673580 10.1016/j.artmed.2023.102617

[6] Elkjaer AS, Lynge SK, Grauslund J. Evidence and indications for systemic treatment in diabetic retinopathy: a systematic review. Acta Ophthalmol. 2020;98(4):329‐336. 32100477 10.1111/aos.14377

[7] Bansal N . Prediabetes diagnosis and treatment: a review. World J Diabetes. 2015;6(2):296. 25789110 10.4239/wjd.v6.i2.296 PMC4360422

[8] Uusitupa M, Khan TA, Viguiliouk E, et al Prevention of type 2 diabetes by lifestyle changes: a systematic review and meta-analysis. Nutrients. 2019;11(11):2611. 31683759 10.3390/nu11112611 PMC6893436

[9] Gong Q, Gregg EW, Wang J, et al Long-term effects of a randomised trial of a 6-year lifestyle intervention in impaired glucose tolerance on diabetes-related microvascular complications: the China Da Qing Diabetes Prevention Outcome Study. Diabetologia. 2011;54(2):300‐307. 21046360 10.1007/s00125-010-1948-9

[10] Munch IC, Kessel L, Borch-Johnsen K, Glümer C, Lund-Andersen H, Larsen M. Microvascular retinopathy in subjects without diabetes: the Inter99 Eye Study. Acta Ophthalmol. 2012;90(7):613‐619. 21470389 10.1111/j.1755-3768.2011.2148.x

[11] Aro A, Kauppinen A, Kivinen N, et al Life style intervention improves retinopathy Status-the Finnish diabetes prevention study. Nutrients. 2019;11(7):1691. 31340493 10.3390/nu11071691 PMC6683279

[12] Sune MP, Sune M, Sune P, Dhok A. Prevalence of retinopathy in prediabetic populations: a systematic review and meta-analysis. Cureus. 2023;15(11):e49602. 38161917 10.7759/cureus.49602 PMC10755086

[13] White NH, Pan Q, Knowler WC, et al Risk factors for the development of retinopathy in prediabetes and type 2 diabetes: the diabetes prevention program experience. Diabetes Care. 2022;45(11):2653‐2661. 36098658 10.2337/dc22-0860 PMC9679265

[14] Tuomilehto J, Lindström J, Eriksson JG, et al Prevention of type 2 diabetes mellitus by changes in lifestyle among subjects with impaired glucose tolerance. N Engl J Med. 2001;344(18):1343‐1350. 11333990 10.1056/NEJM200105033441801

[15] Lindström J, Louheranta A, Mannelin M, et al The Finnish Diabetes Prevention Study (DPS): lifestyle intervention and 3-year results on diet and physical activity. Diabetes Care. 2003;26(12):3230‐3236. 14633807 10.2337/diacare.26.12.3230

[16] Lindström J, Peltonen M, Eriksson JG, et al Finnish diabetes Prevention Study (DPS). Improved lifestyle and decreased diabetes risk over 13 years: long-term follow-up of the randomised Finnish Diabetes Prevention Study (DPS). Diabetologia. 2013;56(2):284‐293. 23093136 10.1007/s00125-012-2752-5

[17] de Mello VD, Lindström J, Eriksson J, et al Insulin secretion and its determinants in the progression of impaired glucose tolerance to type 2 diabetes in impaired glucose-tolerant individuals: the Finnish Diabetes Prevention Study. Diabetes Care. 2012;35(2):211‐217. 22210578 10.2337/dc11-1272 PMC3263888

[18] de Mello VD, Selander T, Lindström J, Tuomilehto J, Uusitupa M, Kaarniranta K. Serum levels of plasmalogens and fatty acid metabolites associate with retinal microangiopathy in participants from the Finnish diabetes prevention study. Nutrients. 2021;13(12):4452. 34960007 10.3390/nu13124452 PMC8703764

[19] Eriksson J, Lindström J, Valle T, et al Prevention of type II diabetes in subjects with impaired glucose tolerance: the Diabetes Prevention Study (DPS) in Finland. Diabetologia. 1999;42:793‐801. 10440120 10.1007/s001250051229

[20] Gould AL, Boye ME, Crowther MJ, et al Joint modeling of survival and longitudinal non-survival data: current methods and issues. Report of the DIA Bayesian joint modeling working group. Stat Med. 2015;34(14):2181‐2195. 24634327 10.1002/sim.6141 PMC4677775

[21] Brown ER . Assessing the association between trends in a biomarker and risk of event with an application in pediatric HIV/AIDS. Ann Appl Stat. 2009;3(3):1163‐1182. 20802852 10.1214/09-aoas251 PMC2928653

[22] Rizopoulos D . Joint Models for Longitudinal and Time-to-Event Data With Applications in R. Chapman and Hall/CRC Biostatistics Series: Boca Raton; 2012.

[23] Kerrison G, Gillis RB, Jiwani SI, et al The effectiveness of lifestyle adaptation for the prevention of prediabetes in adults. A systematic review. J Diabetes Res. 2017;2017:8493145. 28567425 10.1155/2017/8493145 PMC5439262

[24] Thipsawat S . Intervention for prevention of type 2 diabetes Mellitus among prediabetes: a review of the literature. SAGE Open Nurs. 2023;9:23779608231175581. 37324573 10.1177/23779608231175581 PMC10265340

[25] Chen Y, Zhang P, Wang J, et al Associations of progression to diabetes and regression to normal glucose tolerance with development of cardiovascular and microvascular disease among people with impaired glucose tolerance: a secondary analysis of the 30 year Da Qing Diabetes Prevention Outcome Study. Diabetologia. 2021;64(6):1279‐1287. 33608769 10.1007/s00125-021-05401-x

[26] Gong Q, Zhang P, Wang J, et al Morbidity and mortality after lifestyle intervention for people with impaired glucose tolerance: 30-year results of the Da Qing Diabetes Prevention Outcome Study. Lancet Diabetes Endocrinol. 2019;7(6):452‐461. 31036503 10.1016/S2213-8587(19)30093-2 PMC8172050

[27] Gong Q, Zhang P, Wang J, et al Efficacy of lifestyle intervention in adults with impaired glucose tolerance with and without impaired fasting plasma glucose: a post hoc analysis of Da Qing Diabetes Prevention Outcome Study. Diabetes Obes Metab. 2021;23(10):2385‐2394. 34212465 10.1111/dom.14481 PMC8429240

[28] Díaz-López A, Babio N, Martínez-González MA, et al Mediterranean diet, retinopathy, nephropathy, and microvascular diabetes complications: a post hoc analysis of a randomized trial. Diabetes Care. 2015;38(11):2134‐2141. 26370380 10.2337/dc15-1117

[29] Diabetes Prevention Program Research Group. Long-term effects of lifestyle intervention or metformin on diabetes development and microvascular complications over 15-year follow-up: the Diabetes Prevention Program Outcomes Study. Lancet Diabetes Endocrinol. 2015;3(11):866‐875. 26377054 10.1016/S2213-8587(15)00291-0 PMC4623946

[30] Larsen MB, Henriksen JE, Grauslund J, Peto T. Prevalence and risk factors for diabetic retinopathy in 17 152 patients from the island of Funen, Denmark. Acta Ophthalmol. 2017; 95(8):778‐786. 28444837 10.1111/aos.13449

